# Tracing the origins and signatures of selection of antifolate resistance in island populations of *Plasmodium falciparum*

**DOI:** 10.1186/1471-2334-10-163

**Published:** 2010-06-09

**Authors:** Patrícia Salgueiro, José L Vicente, Conceição Ferreira, Vânia Teófilo, André Galvão, Virgílio E do Rosário, Pedro Cravo, João Pinto

**Affiliations:** 1 UEI Malária & UEI Biologia Molecular, Centro de Malária e outras Doenças Tropicais, Instituto de Higiene e Medicina Tropical, Universidade Nova de Lisboa. Rua da Junqueira 100, 1349-008 Lisboa, Portugal; 2 Centro Nacional de Endemias, Ministério da Saúde. Caixa Postal 218, São Tomé, República Democrática de São Tomé e Príncipe

## Abstract

**Background:**

Resistance of the malaria parasite *Plasmodium falciparum *to sulfadoxine-pyrimethamine (SP) has evolved worldwide. In the archipelago of São Tomé and Principe (STP), West Africa, although SP resistance is highly prevalent the drug is still in use in particular circumstances. To address the evolutionary origins of SP resistance in these islands, we genotyped point mutations at *P. falciparum dhfr *and *dhps *genes and analysed microsatellites flanking those genes.

**Methods:**

Blood samples were collected in July and December 2004 in three localities of São Tomé Island and one in Principe Island. Species-specific nested-PCR was used to identify *P. falciparum *infected samples. Subsequently, SNPs at the *dhfr *and *dhps *genes were identified through PCR-RFLP. Isolates were also analysed for three microsatellite loci flanking the *dhfr *gene, three loci flanking *dhps *and four loci located at putative neutral genomic regions.

**Results:**

An increase of resistance-associated mutations at *dhfr *and *dhps *was observed, in particular for the *dhfr*/*dhps *quintuple mutant, associated with clinical SP failure. Analysis of flanking microsatellites suggests multiple independent introductions for *dhfr *and *dhps *mutant haplotypes, possibly from West Africa. A reduced genetic diversity and increased differentiation at flanking microsatellites when compared to neutral loci is consistent with a selective sweep for resistant alleles at both loci.

**Conclusions:**

This study provides additional evidence for the crucial role of gene flow and drug selective pressures in the rapid spread of SP resistance in *P. falciparum *populations, from only a few mutation events giving rise to resistance-associated mutants. It also highlights the importance of human migration in the spread of drug resistant malaria parasites, as the distance between the islands and mainland is not consistent with mosquito-mediated parasite dispersal.

## Background

Resistance to virtually all classes of antimalarial drugs has evolved in natural populations of *Plasmodium falciparum*, the human malaria parasite species responsible for the great majority of malaria-attributed child mortality and morbidity. Resistance to chloroquine (CQ) is prevalent in nearly all malaria-endemic regions, leading to the discontinued use of this drug globally. Resistance to sulfadoxine-pyrimethamine (SP), an affordable and widely available alternative to CQ, has also spread rapidly. Furthermore, therapeutic failure to the most recent arteminisin combination therapies (ACTs) has been reported in Southeast Asia [1].

Mutations in genes encoding dihydrofolate reductase (*dhfr*) and dihydropteroate synthase (*dhps*) enzymes of the parasite's folate pathway are associated with resistance to pyrimethamine and sulfadoxine, respectively [2,3]. Point mutations at codons 51, 59 and 108 of *dhfr*, operate synergistically to enhance resistance to pyrimethamine. The level of resistance is much higher in a triple mutant N51I/C59R/S108N than in a single or double mutant. Likewise, mutations at codons 437 and 540 of *dhps *increase the level of resistance to sulfadoxine, with the double mutant A437G/K540E showing the highest level of resistance. Considering the two genes together, the quintuple mutant N51I/C59R/S108N/A437G/K540E accounts for clinical failure of SP [4].

As a result of the increased levels of resistance, SP has been gradually removed from malaria control programs worldwide. Nevertheless, selective pressure for SP-resistant mutants persists, given the continued use of SP in specific situations. Owing to its safety, SP is still recommended for intermittent preventive therapy (IPT) in pregnant women and children, even in regions where SP efficacy for treatment of severe malaria is compromised [5]. Also, an antifolate compound similar to SP, trimethoprim-sulphamethoxazole, is suggested to prevent opportunistic infections in HIV-infected patients in developing countries [6]. Therefore, the origin and evolution of SP resistance remains a topic of both evolutionary and epidemiological relevance. Furthermore, insights on resistance to SP may also contribute to a better understanding of the evolutionary dynamics of resistance to other drugs.

Roper *et al*. [7] presented evidence suggesting that the *dhfr *triple mutant N51I/C59R/S108N occurring in South Africa had a common ancestry with the Southeast Asian triple mutant. Subsequent studies have shown that this haplotype has spread throughout the African continent and only very few local/regional haplotype variants of African origin have been so far reported [8-12]. These studies demonstrate also relatively few independent origins of double mutants at the *dhfr *gene, though greater variation can be found in single mutants. Fewer studies are available for the *dhps *gene but a recent continent-wide study of Sub-Saharan African *P. falciparum *populations has shown that mutant *dhps *alleles have originated from five independent geographically delimited ancestral lineages: three for single mutants (A437G) and two for double mutants (A437G/K540E) [13]. Collectively, the above findings highlight that limited mutation events followed by extensive gene flow have driven SP resistance in *P. falciparum *populations worldwide. Interestingly, this appears not to be the case in *Plasmodium vivax *for which resistance-associated mutations at the *dhfr *locus have arisen from multiple independent mutation events in Southeast Asia [14].

Selection of mutant parasites through drug pressure is also a key factor for the rapid spread of SP resistance-associated alleles. In a situation of marked selective pressure acting on advantageous mutations at a locus, genetic diversity tends to decrease and linkage disequilibrium increases in the surrounding regions through genetic hitchhiking. This process, also known as selective sweep, can be detected by the analysis of putatively neutral genetic markers flanking the locus under selection at increasing distances [15]. Using this approach, selective sweeps of resistant *dhfr *and *dhps *alleles have been described for *P. falciparum *samples from Southeast Asia, Africa and South America [10,16-18].

Until recently, CQ and SP remained respectively the 1^st ^and 2^nd ^line therapeutic lines for malaria in the archipelago of São Tomé and Principe (STP). However, the increasing levels of both *in vivo *and *in vitro *resistance to these antimalarials coupled with molecular surveys pointing to high levels of resistance-associated mutations [19-22], led to a change in the malaria therapeutic regime. In the case of SP resistance genes, two reports point to an increase of triple mutants N51I/C59R/S108N for the *dhfr *gene [21,22]. In 2004, the STP government approved a new antimalarial drug regime with artemisinin-based combination therapies [23]. Since then STP has become one of the few countries where the malaria burden was reduced by 50% or more between 2000 and 2006-2007, in line with WHO targets. Presently, SP is used in the islands only for IPT during pregnancy. In 2007 the rate of IPT use by pregnant women reached 76% [23], indicating that selective pressure for SP-resistant mutants is still present in STP.

In this context, the aims of the present study were: i) to obtain a contemporary snapshot on the prevalence of point mutations in the *pfdhfr *and *pfdhps *genes in natural *P. falciparum *populations of STP; and ii) to investigate the origin and evolutionary dynamics of these mutations, by analysing microsatellite loci flanking these genes. Results are interpreted with respect to the main evolutionary forces driving SP resistance in the local *P. falciparum *populations, in light of the fact that these are island populations, in which gene flow is likely to be more restricted than in the mainland.

## Methods

### Study sites and sampling

The Democratic Republic of São Tomé and Príncipe (STP) is located in the Gulf of Guinea, *ca*. 240 km off the coast of Gabon, West Africa. It comprises two main islands: São Tomé (859 km^2^) and Príncipe (142 km^2^), with most of the 150,000 residents living in São Tomé. Malaria reaches meso-hyperendemic levels and all four human *Plasmodium *species are present in both islands, but with large predominance of *P. falciparum *infections [24].

Blood samples were collected in July 2004 by finger prick from children up to nine years-old, from three localities of São Tomé - Neves (0°22'N; 6°32'E), Ribeira Afonso (0°12'N; 6°43'E) and Angolares (0°4'N; 6°32'E), and one from Príncipe - Rua dos Trabalhadores (1°38'N; 7°25'E). Collections were undertaken within the framework of active-case malariological surveys carried out by the Centro Nacional de Endemias, Ministry of Health of STP. Ethical clearance was obtained from the Ministry of Health of STP. Informed consent was obtained by the parents or tutors of the children sampled. The localities of Neves and Angolares were sampled a second time, in December 2004. Henceforward, these samples will be referred to as Neves II and Angolares II, respectively. Individual blood samples were spotted onto Whatman No. 4 filter papers and kept dry at room temperature until further processing.

### DNA isolation, species identification and genotyping of dhfr and dhps

Blood spot samples were used to perform DNA extraction by a Chelex protocol [25]. Each DNA sample was then used in a *nested*-PCR reaction, in order to identify the presence of *P. falciparum *according to Snounou *et al*. [26]. Using the PCR-RFLP method described by Duraisingh *et al*. [27], parasite DNA was checked for Single Nucleotide Polymorphisms (SNPs) at codons 51, 59 and 108 of the *dhfr *gene and at codons 437 and 540 of the *dhps *gene.

### Genotyping of microsatellite loci

In order to determine the evolutionary origins of *dhfr *and *dhps *haplotypes, we explored polymorphic microsatellites flanking both genes, located 0.3 kb, 4.4 kb and 5.3 kb upstream of codon 108 of *dhfr *(chromosome 4) and 0.8 kb, 4.3 kb and 7.7 kb downstream from codon 437 of *dhps *(chromosome 8). A semi-nested PCR was performed for each locus using the primers originally described by Roper *et al*. [28] but with the primer modifications (*i.e*. nucleotide extensions) suggested by Ndiaye *et al*. [11] for the *dhfr *flanking microsatellites, to minimise stuttering. PCR reactions and cycling conditions were as described by Roper *et al*. [28]. Amplified fragments were separated by capillary electrophoresis in an automatic sequencer (ABI 3730, Applied Biosystems) at the Yale University DNA Analysis Facility on Science Hill. Allele sizes were scored using the software GeneMarker^® ^(SoftGenetics). As a reference, we have used the Southeast Asian *P. falciparum *K1 laboratory strain. This strain has a known microsatellite profile for the loci used that differs from the widespread *dhfr *triple mutant lineage of Asian origin (Roper C., pers. comm.). At microsatellites flanking the *dhps *gene, the allelic composition of the K1 strain matches that of the East African *dhps *double mutant A437G/K540E haplotype lineage SGE 1 [13].

As suggested by McCollum *et al*. [10], we considered a *dhfr *or *dhps *allele sensitive if it lacked drug resistance-associated mutations within the coding region. The term "haplotype" refers to a particular multilocus genotype composed by the resistance-specific polymorphic locus and the corresponding flanking microsatellite loci. Haplotypes were differentiated if they contained one or more allelic change across the loci. For haplotype reconstruction, we excluded all samples that failed to amplify in any of the loci or that generated multiple peaks [28], in order to avoid incorrect haplotype calling. For the remaining analyses, if multiple peaks (alleles) were detected at any locus, only the predominant allele (*i.e*. the peak with the highest amplification intensity) was chosen as the representative haplotype.

In order to obtain a measure of genetic variation and linkage disequilibrium at putative neutral regions, we genotyped further four microsatellite loci: TAA81 (chr. 5), PfPK2 (chr. 12), TAA109 and TAA87 (chr. 6). Primer sequences and PCR conditions are described in [29,30]. PCR products were separated and scored as described above. For each microsatellite locus, in cases where multiple peaks were present, only the value of the highest peak was scored. The results derived from these neutral markers were put in context with the patterns observed in the microsatellites surrounding the *dhfr *and *dhps *genes.

### Statistical analysis

We assessed allelic richness (*R*_*s*_), a measure of the number of alleles independent of sample size, using FSTAT v. 2.9.3 [31]. The Microsoft^® ^Office Excel Microsatellite tool kit [32] was used to obtain allele frequencies for expected heterozygosity (*H*_*e*_) calculations. We calculated *H*_*e *_as in Anderson *et al*. [33]. Genetic differentiation per locus in the total population sample was measured using an unbiased estimator of *F*_*ST *_[34] available in FSTAT. Linkage disequilibrium (LD) was computed by exact tests of LD available in GENEPOP v. 4.0 [35]. Initially, we compared sulfadoxine-sensitive and resistant genotypes at the *dhps *locus by defining subsamples as wild-type (or sensitive), single mutant (A437G) or double mutant (A437G/K540E). This approach was not implemented for the pyrimethamine resistance-associated *dhfr *gene, because we did not detect any wild-type individuals and the number of single mutants was extremely low. Therefore, we could only compare triple mutants (N51I/C59R/S108N) with double mutants (N51I/S108N). Corrections of the nominal significance value (α = 0.05) to account for multiple testing were performed by a sequential Bonferroni procedure [36].

## Results

### Point mutations at dhfr and dhps loci

Of the 934 blood samples analysed, approximately one third (*N *= 326) were positive for *P. falciparum*. Table 1 describes the prevalence of wild-type, mutant and mixed alleles infections for both *dhfr *and *dhps *genes, as well as the corresponding genotypes. Allelic frequencies were overall similar when comparing different sampling sites and years.

**Table 1:** Allele and genotype frequencies of *dhfr and dhps *genes in *Plasmodium falciparum of *STP

Island	Site	Mutations						Genotypes							
		
			***dhfr***			***dhps***		***dhfr***				***dhps***			***dhfr/dhps***
		
			N51I	C59R	S108N	A437G	K540E	WT	single	double	triple	WT	single	double	quintuple
Príncipe	Rua dos Trabalhadores	wild type	0.00	0.43	0.00	0.00	0.88								
		mutant	1.00	0.57	1.00	0.88	0.13	0.00	0.00	0.50	0.50	0.00	0.88	0.13	0.00
		mixed	0.00	0.00	0.00	0.13	0.00								
		*N*	8	7	9	8	8	6				8			5

S. Tomé	Ribeira Afonso	wild type	0.00	0.36	0.00	0.05	0.90								
		mutant	0.97	0.56	0.97	0.82	0.05	0.00	0.00	0.32	0.68	0.05	0.86	0.10	0.18
		mixed	0.03	0.08	0.03	0.14	0.05								
		*N*	29	25	29	22	21	19				21			11
	Neves	wild type	0.08	0.48	0.00	0.23	1.00								
		mutant	0.89	0.44	1.00	0.67	0.00	0.00	0.00	0.55	0.45	0.23	0.77	0.00	0.00
		mixed	0.03	0.07	0.00	0.10	0.00								
		*N*	37	27	31	30	32	22				30			18
	Neves II	wild type	0.00	0.39	0.05	0.11	0.98								
		mutant	0.96	0.57	0.93	0.80	0.00	0.00	0.00	0.40	0.60	0.10	0.88	0.02	0.00
		mixed	0.04	0.04	0.01	0.09	0.02								
		*N*	73	46	76	64	60	40				60			30
	Angolares	wild type	0.02	0.30	0.00	0.18	0.84								
		mutant	0.98	0.60	1.00	0.80	0.09	0.00	0.03	0.29	0.66	0.18	0.66	0.16	0.09
		mixed	0.00	0.10	0.00	0.02	0.07								
		*N*	41	50	47	44	45	38				44			33
	Angolares II	wild type	0.00	0.18	0.03	0.07	0.78								
		mutant	1.00	0.73	0.93	0.89	0.21	0.00	0.00	0.20	0.80	0.07	0.71	0.22	0.19
		mixed	0.00	0.08	0.04	0.04	0.02								
		*N*	63	60	71	55	58	50				55			32

STP	All samples	wild type	0.02	0.32	0.02	0.12	0.89								
		mutant	0.96	0.60	0.96	0.81	0.08	0.00	0.01	0.33	0.66	0.12	0.78	0.11	0.09
		mixed	0.02	0.07	0.02	0.07	0.03								
		*N*	251	215	263	223	224	175				218			129

*N*: total number of alleles or genotypes

For *dhfr*, pyrimethamine resistance-associated mutations N51I and S108N were extremely predominant (96%) and 60% of the samples harboured the C59R mutation. No wild-type genotypes were found and only one sample harboured a single mutation. The triple mutant N51I/C59R/S108N was present in 66% of the samples. As for *dhps*, the most frequent point mutation was A437G (81%). The double *dhps *mutant A437G/K540E appeared in 11% of the samples. Eleven (9%) of the samples harboured the quintuple *dhfr/dhps *mutant (N51I/C59R/S108N/A437G/K540E), which is predictive of clinical resistance to SP [4].

### dhfr and dhps haplotype characterization

Haplotypes were characterized only in samples for which no multiple infections were detected or that had not failed PCR amplification in any of the loci. The frequency of polyclonal infections was high, as expected from the meso-hyperendemic levels of malaria transmission characteristic of STP [24]. Consequently, our sample size was considerably reduced for this analysis, with haplotypes being reconstructed in 23 out of 175 genotyped isolates for *dhfr *and in 9 out of 218 genotyped isolates for *dhps*.

For the *dhfr *gene, we detected five distinct haplotypes classified as H1 to H5 according to the allele sizes at the 0.3 kb, 4.4 kb and 5.3 kb loci and the mutations carried (Table 2). Three of these haplotypes were associated with the triple mutant N51I/C59R/S108N and the two remaining corresponded to double mutants N51I/S108N. Haplotypes H2 and H3 had the same microsatellite composition but carried double and triple *dhrf *mutations, respectively. The most abundant haplotype associated with the triple mutant (H5), consisting of 114bp/183bp/210 bp at the 0.3 kb/4.4 kb/5.3 kb loci respectively, has matching microsatellite sizes with the *P. falciparum *K1 laboratory strain used as a control. We did not find any parasite carrying the wild-type allele at the three point mutations of the *dhfr *gene, which precluded the identification of haplotypes associated with pyrimethamine sensitive parasites.

**Table 2:** *dhfr *point mutations and their respective microsatellite haplotypes in allele size

Haplotype	Point mutations	Allele size (basepairs)			*N*
			
		Locus 0.3 kb	Locus 4.4 kb	Locus 5.3 kb	
**K1**	**C59R/S108N**	**114**	**183**	**210**	-
H1	N51I/S108N	108	181	210	2
H2	N51I/S108N	108	183	200	10
H3	N51I/C59R/S108N	108	183	200	1
H4	N51I/C59R/S108N	108	183	210	1
H5	N51I/C59R/S108N	114	183	210	9

K1: reference isolate (see methods). *N*: number of isolates with the correspondent haplotype.

Regarding *dhps*, eight distinct haplotypes were detected and classified as H1 to H8 according to allele sizes at 0.8 kb, and 4.3 kb and 7.7 kb loci (Table 3). Five haplotypes were associated with the single mutant A437G. The *dhps *double mutant A437G/K540E was linked to haplotype H7 consisting of 131 bp/103 bp/108 bp at the 0.8 kb/4.3 kb/7.7 kb loci respectively. This haplotype is an exact match of the K1 control. Two haplotypes, H5 and H8, were associated with wild type alleles.

**Table 3:** *dhps *point mutations and their respective microsatellite haplotypes in allele size

Haplotype	Point mutations	Allele size (basepairs)			*N*
			
		Locus 0.8 kb	Locus 4.3 kb	Locus 7.7 kb	
**K1**	**A437G**	**131**	**103**	**108**	-
H1	A437G	117	105	104	1
H2	A437G	117	105	106	1
H3	A437G	117	105	116	1
H4	A437G	117	109	134	1
H5	Wild type	123	103	110	1
H6	A437G	123	103	124	1
H7	A437G/K540E	131	103	108	2
H8	Wild type	133	117	110	1

K1: reference isolate (see methods). *N*: number of isolates with the correspondent haplotype.

### Genetic variation and linkage disequilibrium

The effect of SP selection in the *P. falciparum *population of STP was assessed by examining the levels of genetic diversity, linkage disequilibrium and genetic differentiation in the microsatellite loci flanking the *dhfr *and *dhps *genes, in comparison with neutral loci. We expected to observe signatures of selection left by SP pressure in this population [37], namely: i) a reduction in genetic diversity around *dhps *and *dhfr*, due to a selective sweep; ii) an increased genetic differentiation at the loci under selection; iii) significant LD between loci flanking *dhps *and *dhfr *genes.

Overall, genetic diversity estimates at neutral loci (*H*_*e *_= 0.82; *R*_*s *_= 6.5; *N *= 308) were higher than at loci flanking *dhfr *(*H*_*e *_= 0.64; *R*_*s *_= 4.6; *N *= 298) and *dhps *(*H*_*e *_= 0.74; *R*_*s *_= 5,1; *N *= 296) genes. For neutral loci, a low but significant *F*_*ST *_of 0.016 (*P *< 0.001) was obtained for the whole sample subdivided according to collection sites, suggesting a relatively shallow substructuring of *P. falciparum *populations among the localities surveyed. Differentiation was however higher at flanking microsatellites, with *F*_*ST *_values of 0.043 (*P *< 0.001) and 0.027 (*P *< 0.001) for *dhfr *and *dhps-*linked loci, respectively.

In parasites harbouring *dhfr *resistant alleles (double and triple mutants), microsatellites flanking the *dhfr *gene had lower genetic variation than neutral loci and loci flanking the *dhps *gene (Table 4). Also, genetic differentiation was higher at *dhfr *flanking loci, mainly attributed to loci 0.3 kb (*F*_*ST *_= 0.38) and 5.3 kb (*F*_*ST *_= 0.57) when compared to neutral loci (-0.01 ≤ *F*_*ST *_≤ 0.02) or to loci flanking the *dhps *gene (-0.01 ≤ *F*_*ST *_≤ 0.01). The levels of genetic diversity and differentiation were similar in neutral loci and in loci flanking the *dhps *gene.

**Table 4:** Allele richness (*R*_*s*_), expected heterozygosity (*H*_*e*_) and *F*_*S*__*T *_estimates at flanking and neutral microsatellites of *P. falciparum dhfr *double and triple mutants

		*dhfr *genotypes							
		
Microsatellites		Double mutant (*N *= 52)		Triple mutant (*N *= 99)		all samples (*N *= 151)			
		*R*_*s*_	*H*_*e*_	*R*_*s*_	*H*_*e*_	*R*_*s*_	*H*_*e*_	*F*_*ST*_	*P*

**Loci flanking *dhfr *gene**	*Dhfr *0.3	8	0.34	7	0.68	9	0.51	0.38	**<0.0001**
	*Dhfr *4.4	8	0.48	6	0.45	7	0.46	-0.01	NS
	*Dhfr *5.3	6	0.35	6	0.37	7	0.36	0.57	**<0.0001**
	All loci	7	0.39	6	0.50	8	0.44	0.37	**<0.0001**
**Loci flanking *dhps *gene**	*Dhps *0.8	12	0.67	10	0.53	13	0.60	0.01	NS
	*Dhps *4.3	8	0.70	7	0.67	8	0.69	0.01	NS
	*Dhps *7.7	14	0.92	15	0.90	17	0.91	-0.01	NS
	All loci	11	0.76	11	0.70	13	0.73	0.00	NS
**Neutral loci**	TAA81	8	0.83	11	0.84	10	0.83	0.02	NS
	TA109	10	0.76	11	0.72	11	0.74	0.01	NS
	TA87	11	0.89	12	0.85	12	0.87	0.01	NS
	PfPK2	10	0.85	13	0.88	13	0.87	-0.01	NS
	All loci	10	0.83	12	0.82	12	0.83	0.01	NS

*N*: Number of isolates genotyped. All loci: mean over loci *R*_*s *_and *H*_*e *_and global *F*_*ST *_over loci as calculated by FSTAT. *P: P-*values of permutation tests to assess significance of *F*_*ST *_values. NS: non-significant (*P *> 0.05). In bold: significant after Bonferroni corrections for multiple tests.

Microsatellite estimates for parasites carrying wild-type or resistance-associated alleles at the *dhps *loci are shown in Table 5. Mutant parasites, particularly double mutants, presented lower levels of genetic diversity than wild-type ones at flanking loci. Among wild-type alleles, genetic diversity at flanking loci was comparable to that observed for neutral loci. Genetic differentiation was higher at loci that flanked the *dhps *gene (0.12 ≤ *F*_*ST *_≤ 0.30) when compared to neutral (-0.01 ≤ *F*_*ST *_≤ 0.02) or *dhfr-*linked (0.00 ≤ *F*_*ST *_≤ 0.01) loci.

**Table 5:** Allele richness (*R*_*s*_), expected heterozygosity (*H*_*e*_) and *F*_*S*__*T *_estimates at flanking and neutral microsatellites of *P. falciparum dhps *genotypes

		*dhps *genotypes									
		
Microsatellites		Wild type (*N *= 26)		Single mutant (*N *= 154)		Double mutant (*N *= 15)		all samples (*N *= 195)			
		*R*_*s*_	*H*_*e*_	*R*_*s*_	*H*_*e*_	*R*_*s*_	*H*_*e*_	*R*_*s*_	*H*_*e*_	*F*_*ST*_	*P*

**Loci flanking *dhps *gene**	Dhps 0.8	8	0.91	4	0.47	5	0.79	6	0.71	0.25	**<0.0001**
	Dhps 4.3	6	0.85	4	0.53	3	0.26	4	0.55	0.30	**<0.0001**
	Dhps 7.7	8	0.90	10	0.92	3	0.28	9	0.71	0.12	0.02
	Over all loci	7	0.82	6	0.63	4	0.45	6	0.66	0.22	**<0.0001**
**Loci flanking *dhfr *gene**	*Dhfr *0.3	5	0.68	6	0.72	5	0.76	6	0.72	0.02	NS
	*Dhfr *4.4	4	0.49	5	0.44	2	0.13	5	0.36	<-0.01	NS
	*Dhfr *5.3	5	0.66	5	0.63	3	0.53	5	0.61	-0.01	NS
	Over all loci	5	0.61	5	0.60	3	0.47	5	0.56	0.01	NS
**Neutral loci**	TAA81	7	0.84	7	0.83	6	0.83	7	0.83	0.01	NS
	TA109	6	0.74	8	0.77	4	0.50	8	0.67	0.01	NS
	TA87	9	0.87	9	0.87	7	0.87	9	0.87	<0.01	NS
	PfPK2	7	0.85	10	0.88	6	0.86	10	0.86	0.01	NS
	Over all loci	7	0.83	9	0.84	6	0.77	9	0.81	0.01	NS

*N*: Number of isolates genotyped. All loci: mean over loci Rs and He and global *F*_*ST *_over loci as calculated by FSTAT. *P: P-*values of permutation tests to assess significance of *F*_*ST *_values. NS: non-significant (*P *> 0.05). In bold: significant after Bonferroni corrections for multiple tests.

Exact tests of linkage disequilibrium revealed three significant associations between pairs of microsatellite loci out of 92 tests performed with isolates carrying *dhfr *double or triple mutants. Of these, there was a single significant association between loci flanking the *dhfr *gene (loci 4.4 kb and 5.3 kb) that was found in the sample of double mutants. For the *dhps *gene, there were 6 significant pairwise associations between microsatellite loci out of 135 tests. These were all detected in the group of single mutants and there was also only a single significant association involving two microsatellites flanking the *dhps *gene (loci 0.8 kb and 4.3 kb).

## Discussion

When the results of the present study (collections in 2004) are interpreted in light of reports from surveys carried out in 2000 [21] and in 2002 [22], a general trend stands out for an increase in the frequency of mutant parasites, especially of *dhfr *triple mutants and *dhps *double mutants. In 2000, the most common mutations found in STP were N51I (92%) and S108N (95%) for *dhfr*, and A437G (75%) for *dhps *[21]. Our present results support a trend for fixation of the most common mutations at the *dhfr *gene (96% for both N51I and S108N). Regarding the *dhps *gene, the proportion of A437G mutations here reported (81%) is also higher than in 2000 (75%) but lower than in 2002 (86%) [22]. In contrast, only 7% of *P. falciparum *isolates harboured the K540E mutation in 2000 [21] and none of the isolates carried this mutation in 2002 [22]. This could have been due to a smaller sample size or a more restricted geographic sampling area in the latter study. In the present dataset the frequency of the *dhps *K540E was 11% (8% single + 3% mixed infections, Table 1) and there was an increase of the double *dhps *mutant A437G/K540E (2000: <7%; 2004: 11%), consequently leading to a 2-fold rise of the *dhfr*+*dhps *quintuple mutant (2000: 4%; 2004: 9%). The K540E mutation is only seldomly found in West Africa [13]. In contrast, in East Africa both the K540E allele and its associated quintuple mutant have reached high prevalence and have been shown to be robust predictors of SP clinical failure [5,38,39]. Therefore, SP remains partially effective in Central Africa possibly due to the still reduced frequency of the K540E mutation. The observed increase in the prevalence of the *dhps *K450E mutation and consequently of the quintuple mutant in STP should thus be considered a subject of concern.

In the present study, two distinct haplotypes were found associated with the double mutant N51I:S1018N and three were associated with the triple mutant N51I:C59R:S1018N. In the triple mutants, the most abundant haplotype, H5 (0.3 kb/4.4 kb/5.3 kb: 114 bp/183 bp/210 bp), is an exact match of the haplotype of the *P. falciparum *K1 laboratory strain. This strain has a microsatellite profile that differs from the intercontinental widespread triple mutant haplotype of Southeast Asian origin (Roper C. pers. comm.). By reviewing the literature, we found haplotypes very close to H5 in Senegal (113 bp/183 bp/211), [11]). In addition, haplotype H4 (108 bp/183 bp/210 bp), that occurred once in our sample, nearly matches the Senegalese haplotype (109 bp/183 bp/211 bp) that was confirmed to be of Southeast Asian origin [11]. Haplotype H3 is likely to have derived from H4 by recombination or mutation as it differs only at the most distal microsatellite locus (5.3 kb). However, the H3 triple mutant may also have arise from H2 double mutants through an additional mutation occurring at position 59 of the *dhfr *gene. Indeed, haplotype H2 shares the same microsatellite profile as H3 and it was the most frequent haplotype in double mutants. Taken together, it appears that *dhfr *triple mutants in STP derive from two independent lineages that were introduced in the islands from continental Africa, although we cannot fully exclude one additional independent local origin associated with haplotype H3.

Similarly, microsatellite haplotype composition for the *dhps *gene provides evidence for at least three independent introductions of mutant parasites in STP. The only *dhps *double mutant haplotype identified has a microsatellite profile coincident with the SGE 1 lineage described by Pearce *et al*. [13]. This double mutant lineage has originated in East Africa where it is the most prevalent mutant lineage. In West Africa, the SGE 1 is rare but it has been detect in a few West African countries, including Gabon in the Gulf of Guinea [13]. In West Africa, A437G single mutants prevail and three independent ancestry lineages have been identified based on the two microsatellites most proximal to the *dhps *gene, 0.8 kb and 4.3 kb [13]. Using the same criterion, haplotypes H1, H2 and H3 can be considered as representatives of the AGK/SGK 1 lineage. This single mutant lineage is the most prevalent in central and Southwest Africa, including Angola, Congo and Gabon [13]. Haplotype H6 has a microsatellite profile closer to the AGK/SGK 3 lineage that displays highest frequencies in countries bordering the Gulf of Guinea such as Cameroon, Gabon, Ghana and Nigeria [13]. Therefore, the haplotype composition of *dhps *mutant parasites found in STP agrees with a scenario of parasite introduction from neighbouring countries of the Gulf of Guinea.

As reported for other *P. falciparum *populations in continental Africa [10,13,17], the analysis of flanking microsatellites provided evidence of a selective sweep for both *dhfr *and *dhps *resistant mutants. Reduced genetic diversity and increased genetic differentiation was observed at microsatellites flanking *dhfr *and *dhps *when compared to neutral loci or when comparing resistant parasites with wild type ones in the case of *dhps*. In the latter, this was more evident for the case of double mutants. These data correlate well with the trend for an increase of *dhps *double mutants since 2000 [21], and consequently of *dhfr/dhps *quintuple mutants.

Population structure could also contribute to the observed patterns of differentiation at flanking loci, particularly if one takes into account that this study analysed *P. falciparum *isolates from two islands. However, when the samples were grouped according to collection site, the *F*_*ST *_estimate obtained for neutral loci, albeit significant, was *ca*. two-fold lower than those observed for the microsatellites flanking *dhfr *and *dhps *genes. Moreover, low and non-significant *F*_*ST *_values were obtained for all neutral microsatellites when samples were grouped according to their genotypes at both genes. It is therefore unlikely that the observed patterns of differentiation at flanking loci result mainly from population subdivision.

In a scenario of a selective sweep acting on recently introduced multiple haplotypes of a mutation under selection (*i.e*. soft sweeps) one expects a strong LD signal [40]. This is because introduced beneficial mutants carrying new haplotypes cause the polymorphic sites to be in complete LD. Unexpectedly, LD tests provided only weak evidence of increased linkage, with a single significant test for a locus pair at *dhfr *double mutants and another for *dhps *single mutants. This observation may be attributed to the fact that SP-resistance in STP has been prevalent for quite sometime and, consequently, LD around these loci has decayed over time due to the accumulation of mutations and also to the effect of recombination between haplotypes [40]. In STP, the first report of therapeutic failure to SP dates from 2000 [21], but positive selection of mutant *dhfr *and *dhps *through drug pressure most likely pre-dates this first finding. Being the 2^nd ^line for malaria treatment, one expects an increased usage of SP since the early-1990's, when considerable levels of resistance to the 1^st ^line chloroquine had already been reported [19]. Altogether these data point to an early introduction of mutant parasites in STP, possibly during the first half of the 1990's, which were subjected to selective pressure due to an already on-going increase of SP usage in the islands. Presence of the *dhfr *triple mutant in Cameroon was reported for the first time in 1985 [9]. The emergence of *dhps *mutants is considered to be more recent, but previous reports suggest that the emergence of all mutant lineages, including the single mutants of West African origin, may have occurred in the early- to mid-1990's (see [13] and refs. therein). These time-scales are in accordance with that here proposed for the introduction mutant parasites in STP.

## Conclusions

In spite of its geographical isolation, resistance to SP in *P. falciparum *populations of STP islands has emerged from multiple introductions of mutant parasites from the continent, possibly of West African origin. Once introduced in the islands, resistance associated mutants have been subjected to positive selection, resulting in a selective sweep detectable at flanking microsatellites, due to the increased usage of SP. As a consequence, it took little more than a decade for resistant parasites to reach near fixation frequencies. These findings reinforce the crucial role of gene flow and strong selective pressures in the rapid establishment of resistance to SP in *P. falciparum *populations. They also highlight the importance of human migration in the spread of drug resistant malaria parasites, as the distance between islands and continent should render mosquito-mediated parasite dispersal unrealistic. Since 2004, a new ACT-based malaria treatment regime was implemented in STP. The previous experience with SP here reported makes a strong case for a continuous monitoring of parasite susceptibility levels, with particular focus on migrants and travellers from endemic areas.

## Competing interests

The authors declare that they have no competing interests.

## Authors' contributions

PS, AG, JLV and VT carried out the molecular analyses. PS and VT performed the genetic data analysis. PS, JP and PC drafted the manuscript. JP, PC and VER conceived the study. CF carried out blood sample collections. All authors read and approved the final manuscript.

## Pre-publication history

The pre-publication history for this paper can be accessed here:

http://www.biomedcentral.com/1471-2334/10/163/prepub
